# BetaMe: impact of a comprehensive digital health programme on HbA1c and weight at 12 months for people with diabetes and pre-diabetes: study protocol for a randomised controlled trial

**DOI:** 10.1186/s13063-018-2528-4

**Published:** 2018-03-05

**Authors:** Diana Sarfati, Melissa McLeod, James Stanley, Virginia Signal, Jeannine Stairmand, Jeremy Krebs, Anthony Dowell, William Leung, Cheryl Davies, Rebecca Grainger

**Affiliations:** 1 Department of Public Health, University of Otago Wellington, PO Box 7343, Wellington, New Zealand; 2 Biostatistical Group, Dean’s Department, University of Otago Wellington, PO Box 7343, Wellington, New Zealand; 3 Department of Medicine, University of Otago Wellington, PO Box 7343, Wellington, New Zealand; 4 Department of Primary Health Care and General Practice, University of Otago Wellington, PO Box 7343, Wellington, New Zealand; 5 Department of Medicine, University of Auckland, PO Box 1142, Auckland, New Zealand; 6 Tu Kotahi Asthma Trust, Lower Hutt, New Zealand

**Keywords:** Diabetes mellitus, Self-management, mHealth, Mobile, Online, Māori, Pacific, Indigenous

## Abstract

**Background:**

Long-term conditions (LTCs) are the biggest contributor to health loss in New Zealand. The economic cost and burden on the health system is substantial and growing. Self-management strategies offer a potential way to reduce the pressure on health services. This study evaluates a comprehensive self-management programme (the BetaMe programme) delivered by mobile and web-based technologies for people with Type 2 diabetes (T2DM) and pre-diabetes. The primary aim of this study is to evaluate the effectiveness of the BetaMe programme versus usual care among primary care populations in improving the control of T2DM and pre-diabetes, as measured by change in HbA1c and weight over 12 months.

**Methods:**

Participants will be recruited through two primary healthcare organisations and a Māori healthcare provider in New Zealand (*n* = 430). Eligible participants will be 18 to 75 years old, with T2DM or pre-diabetes, with an HbA1c of 41–70 mmol/mol up to 2 years prior to study commencement. Eligible participants who consent to participate will be individually randomised to the control arm (usual care) or intervention arm (usual care and BetaMe). The programme consists of a 16-week core followed by a maintenance period of 36 weeks. It incorporates (1) individualised health coaching, (2) goal setting and tracking, (3) peer support in an online forum and (4) educational resources and behaviour-change tools. The primary outcome measures are change in HbA1c and weight at 12 months. Secondary outcomes are changes in waist circumference, blood pressure, patient activation and diabetes-specific behaviours. All outcomes will be assessed at 4 and 12 months for the total study population and for Māori and Pacific participants specifically. All primary analyses will be based on intention-to-treat. Primary analysis will use linear mixed models comparing mean outcome levels adjusted for initial baseline characteristics at 12 months.

**Discussion:**

This is a randomised controlled trial of a comprehensive self-management intervention for people with diabetes and pre-diabetes. If effective, this programme would allow healthcare providers to deliver an intervention that is person-centred and supports the self-care of people with T2DM, pre-diabetes and potentially other LTCs.

**Trial registration:**

Australian New Zealand Clinical Trials Registry, ID: ACTRN12617000549325. Registered on 19 April 2017.

**Electronic supplementary material:**

The online version of this article (10.1186/s13063-018-2528-4) contains supplementary material, which is available to authorized users.

## Background

Long-term health conditions (LTCs, chronic physical and mental disorders) are by far the largest contributor to health loss in New Zealand (NZ), accounting for 88% of disability-adjusted life years [1]. The economic cost and burden on the health system is substantial and growing [1, 2]. Type 2 diabetes mellitus (T2DM) is one of the most common long-term conditions affecting NZ adults, with a prevalence of around 7% based on glycosylated haemoglobin (HbA1c) data from the 2008/09 NZ Adult Nutrition Survey [3]. Furthermore, the prevalence of T2DM is rapidly increasing in NZ at a rate of 7% per annum [2]. Māori, Pacific and Indian people have particularly high rates of T2DM, with rates up to three times higher than European New Zealanders [2, 3]. Diabetes is associated with multiple long-term complications, higher mortality and substantial healthcare costs [2, 4]. Pre-diabetes is a precursor stage to T2DM with up to 70% eventually progressing on to T2DM; many people with pre-diabetes already have some of the complications of T2DM [5–8]. The estimated prevalence of pre-diabetes in NZ adults is 26% [2].

Self-management measures, such as a healthy diet, regular exercise and weight management, are key to preventing the progression of pre-diabetes to T2DM, controlling T2DM and reducing complications [9–13]. For example, the Diabetes Prevention Program (DPP) study found that a lifestyle intervention resulted in a 58% reduction in incidence of diabetes among a high-risk population without diabetes, and that even short-term reversion to normal glucose control among those with pre-diabetes substantially reduced the likelihood that the person would progress to T2DM [12, 13]. Similarly, those with diabetes who undergo lifestyle interventions tend to have better glycaemic control and improved long-term outcomes [9]. However, many people struggle to initiate and maintain these strategies [14, 15].

Self-management strategies for T2DM have resulted in improvements in glycaemic control [16–18], weight loss [17, 18], patient satisfaction with healthcare [17], self-efficacy [19] and adherence to medication [20]. There is less evidence to support the use of self-management programmes in those with pre-diabetes. One study assessed the outcomes of an Internet-based diabetes prevention programme for people with pre-diabetes and found that there were clinically significant reductions in body weight and HbA1c at 2 years after entry into the programme [21].

The BetaMe Programme is an integrated self-management programme for T2DM and pre-diabetes developed by Melon Health Ltd. [22] with a strong emphasis on engagement, education and activation of patients with LTCs. Its foundations are based in behavioural-change theory using cognitive behavioural theory, motivational interviewing, goal setting, health tracking, reminders and intrinsic rewards to support and encourage positive behaviour change [16, 23, 24]. BetaMe was developed in partnership with a multidisciplinary team including primary care practitioners, Māori and Pacific health providers, and psychologists. The programme incorporates access to expert one-on-one coaching, decision support and peer support, all of which are evidence-based approaches to increasing the effectiveness of self-management support (see Table 1).

**Table 1:** Components of the BetaMe programme and evidence of their effectiveness

Stage	Element	What is provided	Evidence-based for effective self-management
Core only (weeks 1–16)	Health coaches	Shared goal setting, and personalised programme based on that person’s personal goals. Provide regular input, encouragement and support via messaging and fortnightly video or audio meetings	Educational programmes and individual support through personalised coaches has been shown to be effective, with the level of effectiveness dependent on the intensity of the programme [50]. A number of successful interventions have provided access to an ‘expert’, such as a personal trainer or dietician, coupled with support from health professionals [51, 52]
	Health literacy	Fortnightly evidence-based resources and behaviour-change tools delivered in consumer-centred formats (bite sized, simple messages, images and video)	Mobile phones to send reminders or educational information via text, or within applications, have proven beneficial in the management of chronic conditions such as diabetes [53] with positive outcomes relating to glycaemic control and patient satisfaction, [17] self-efficacy [19] medication adherence [20] and as a result of weight loss, reduced transition from pre-diabetes to diabetes [54]
Core and maintenance (weeks 1–52)	Goal tracking	Daily reminders via web-based devices. Daily goal tracking of exercise, happiness, energy levels, food and weekly tracking of weight and waist measure	Goal tracking, such as the regular monitoring of weight or laboratory data has been identified as a key component of successful self-management programmes to achieve weight loss [55], and improved long-term outcomes [56]
	Peer support	Online closed forum, monitored by a registered nurse	Peer support has been successful in improving glycaemic control [57–61] and has been identified by participants, to be the most useful component of a self-management programme [57]

BetaMe is delivered using both mobile and web-based platforms in a 12-month programme with four key components: health coaching, provision of evidence-based resources, peer support and goal tracking. Each component has demonstrated benefits for improved outcomes for people with T2DM or pre-diabetes (summarised in Table 1). The first 16 weeks form the core part of the programme, with the remaining 36 weeks covering the maintenance period (web-based peer support and goal-tracking only).

The BetaMe programme underwent substantial pre-testing, and a pilot in 2015 demonstrated very positive results. In the pilot evaluation, 117 people with pre-diabetes participated from five general practices within one region of New Zealand (the Midlands Health Network). Baseline and follow-up measures at 16 weeks included HbA1c, weight, waist circumference, pre-diabetes status (defined as HbA1c 41–49 mmol/mol) and blood pressure. Of the 117 patients, 108 completed the programme (92%). Of these, 91% reduced their HbA1c, 94% lost weight (mean = 4.2 kg), 87% had reduced waist circumference (mean = 4.2 cm) and 78% had final HbA1c levels below the pre-diabetes range [25].

The feedback from pilot participants was extremely positive. Participants strongly endorsed both the coaching and community aspects of the programme (77% found these the most helpful aspect). Most (84%) reported that they were likely to recommend BetaMe to someone else, while only 2% said they were unlikely to do so. In a recent US review of digital diabetes prevention and self-management solutions in the workplace, the BetaMe programme ranked top for patient engagement and was in the top five overall [26].

In this study, we will undertake a randomised controlled trial to compare the BetaMe programme with usual primary care for patients with T2DM and pre-diabetes, examining the impact on HbA1c and weight as well as waist circumference, blood pressure and a number of self-reported measures of effectiveness at 12 months. This work will address several gaps in the current knowledge base around comprehensive self-management programmes for diabetes. First, most existing studies examine only one part of what is considered to be a self-management programme. For example, a 2011 meta-analysis of 22 trials with 1657 participants concluded that there was evidence that mobile-phone applications led to significant improvements in glycaemic control (a mean reduction in HbA1c of 6 mmol/mol over a mean 6 months’ duration) [16]. However, most of the programmes studied were not comprehensive in that their interventions focussed solely on monitoring reminders or entering glucose measurements; did not involve providers or current clinical practice; or did not personalise the programme to individuals. Second, the current study will address a lack of evidence on the effectiveness of self-management programmes for Māori and Pacific individuals. Third, unlike most other studies that focus on ‘people with diabetes’ or ‘pre-diabetes’ our randomised controlled trial (RCT) will include participants with both pre-diabetes and T2DM, and will measure the effectiveness of the intervention for both groups. This will be more representative of the effectiveness of this intervention in the real clinical setting, and will give a better idea of the relative benefits for those with pre-diabetes compared to those with T2DM.

## Methods

### Aims, design and setting

The primary aim of this study is to evaluate the effectiveness of the BetaMe programme versus usual care among primary care populations in improving the control of T2DM and pre-diabetes, as measured by change in HbA1c and weight over a 12-month period.

The secondary aims, which will be explored using secondary analysis of the data, are to evaluate the effectiveness of the BetaMe programme versus usual care in:

1. Improving HbA1c and weight outcomes at 12 months for Māori and Pacific participants specifically (combined across T2DM and pre-diabetes groups);
2. Improving short-term weight management among those with T2DM and pre-diabetes (at 4 months, both overall and reported for Māori and Pacific participants);
3. Improving other health-related outcomes, including waist circumference, blood pressure, health-related quality of life and self-reported self-management (overall and reported for Māori and Pacific participants)

**Fig. 1:**
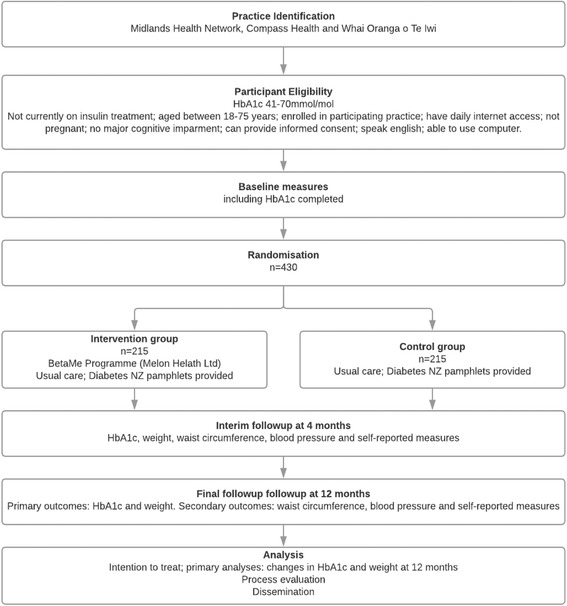
Flowchart of the BetaMe study

**Fig. 2:**
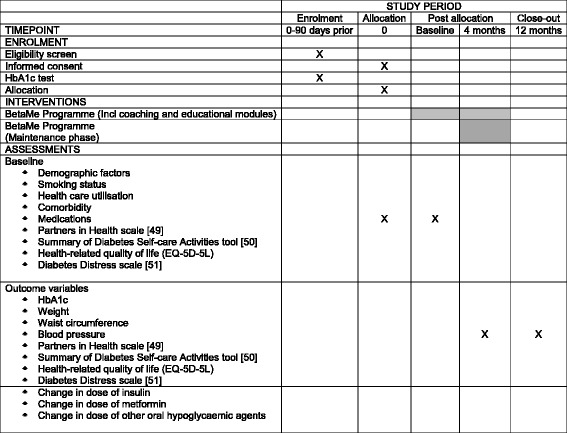
Standard Protocol Items: Recommendations for Interventional Trials (SPIRIT) Figure

The study will be a two-arm, parallel-group, individually randomised controlled trial for people with pre-diabetes and T2DM, comparing the BetaMe programme with usual primary care (active control), with investigator-blinded assessment of outcomes (see Fig. 1 for the flowchart of the BetaMe study, Additional file 1 for the Standard Protocol Items: Recommendations for Interventional Trials (SPIRIT) Checklist and Fig. 2 for the SPIRIT Figure).

The study will be based in two regions of NZ, Midlands and Greater Wellington, through two Primary Healthcare Organisations (PHOs, Midlands Health Network and Compass Health), and a large Māori healthcare provider (Whai Oranga o Te Iwi). These providers collectively include around 800,000 patients. The research team will work directly with the two PHOs to identify and recruit practices that are interested in the study and able to commit to the study timeframes and process requirements. Practice recruitment will be through an opt-in process.

### Participants

Participants with pre-diabetes or T2DM will initially be identified using clinical data held by participating PHOs and practices. Those identified as having T2DM and meeting the eligibility criteria below will be included. Those with HbA1c in the 41–49-mmol/mol range in the 2 years prior to commencing the study, and with no recorded diagnosis of T2DM, will be classified in the pre-diabetes group.

Patients will be eligible for inclusion if they meet the following inclusion criteria:

- Have a current HbA1c of 41–70 mmol/mol (either from a test within 3 months prior to invitation to the study, or on the basis of an HbA1c test taken by the research team at invitation to the study)
- Are not currently on insulin treatment for diabetes
- Adults aged between 18 and 75 years, enrolled in participating practices
- Have daily Internet access (on any of computer/tablet/smartphone)
- Are able to provide informed consent

Patients will not be eligible for the study if they:

- Are pregnant
- Have cognitive impairment likely to be sufficient to hinder their understanding of the study or the programme, as this may negatively affect their self-management behaviour [27]
- Are unable to read and write in English
- Are unable to use phone or computer due to physical disability such as poor eyesight or manual dexterity

### Recruitment

The PHOs or practices will generate a list of current people with T2DM and pre-diabetes, using an HbA1c result of 41–64 mmol/mol within the past 24 months. Individuals that are retested at baseline and return an HbA1c result of 65–70 mmol/mol remain eligible for the study. Patients who are known not to meet the other eligibility criteria will be removed from this list. The remaining patients will be invited by the practice for the research team to contact them.

A research nurse will then contact participants to explain the study, confirm eligibility and obtain informed consent. Baseline data will be collected at this point including HbA1c (for those who do not have an HbA1c test result in preceding 3 months), weight, height, waist circumference and blood pressure (using standardised approaches); along with a health-related quality of life measure, a validated measure of chronic condition self-management; and a validated measure of diabetes-specific behaviours (e.g. frequency of blood sugar monitoring). Details of the measures and questionnaires are in Tables 2 and 3.

**Table 2:** Summary of outcome measures

Outcomes	Time point^a^	Measurement/definition
Primary outcome measures		
Change in HbA1c	At 12 months	Measured using whole blood sample collected in EDTA tube (2 ml minimum) using Variant Turbo Ion Exchange HPLC. Participants will either have a blood sample taken by the research nurse, or will be provided with a laboratory request form for an HbA1c blood test that will be taken as per usual processes within a week of the assessment period.
Change in weight	At 12 months	Measured in kilograms using calibrated digital scales and standardised methods
Secondary outcome measures		
Change in HbA1c	At 4 months	Measured using whole blood sample collected in EDTA tube (2 ml minimum) using Variant Turbo Ion Exchange HPLC (as above)
Change in weight	At 4 months	Measured in kilograms using calibrated digital scales and standardised methods
Change in waist circumference	At 4 and 12 months	Measured in millimetres using tape measure using standardised methods
Change in blood pressure	At 4 and 12 months	Measured using a calibrated syphgmomanometer using a standard approach (blood pressure taken when participant has been sitting quietly for 5 min, without eating, drinking or smoking. They will be asked to have feet flat on the floor, with their back up against the back of the chair, and their left arm straight on the table). Three measures will be taken with the lowest of the last two measures recorded
Change in self-management	At 4 and 12 months	Measured using the Partners in Health Scale [37]
Change of score in diabetic-specific behaviours and outcomes	At 4 and 12 months	Measured using the Summary of Diabetes Self-Care Activities tool, which assess participants’ self-care activities [38]
Health-related quality of life	At 4 and 12 months	Measured using the five-level EuroQol five dimensions questionnaire (EQ-5D-5 L)
Change in score of diabetes-specific outcomes	At 4 and 12 months	Measured using the Diabetes Distress Scale [39]
Change in dose of insulin	At 4 months relative to baseline, and 12 months relative to 4 months	Categorised as (1) starting insulin in current time period; (2) increasing dose of insulin in current period; (3) reducing dose of insulin in current period or (4) stopping insulin in current period. Data will be collected from patients, verified where possible from clinic records
Change in dose of metformin	At 4 months relative to baseline, and 12 months relative to 4 months	Categorised as (1) starting metformin in current time period; (2) increasing dose of metformin in current period; (3) reducing dose of metformin in current period or (4) stopping metformin in current period. Data will be collected from patients, verified where possible from clinic records
Change in dose of other oral hypoglycaemic agents (not metformin)	At 4 months relative to baseline, and 12 months relative to 4 months	Categorised as (1) starting oral hypoglycaemic agents in current time period; (2) increasing dose of oral hypoglycaemic agents in current period; (3) reducing dose of oral hypoglycaemic agents in current period or (4) stopping oral hypoglycaemic agents in current period. Data will be collected from patients, verified where possible from clinic records

*EDTA* ethylenediaminetetracetic acid, *HbA1c* glycosylated haemoglobin, *HPLC* high-performance liquid chromatography

^a^All time periods are time after recruitment, and compared with baseline with exception of changes in medication dose specified above

**Table 3:** Summary of variables measured in the patient questionnaire

Measure	Details	Source
Baseline patient demographic questions	Name, address, date of birth, ethnicity	All questions are based upon Statistics New Zealand census questions: available from: http://archive.stats.govt.nz/methods/classifications-and-standards/classification-related-stats-standards/gender-identity.aspx?_ga=2.83660500.398110315.1519357080-701333020.1444617687
Smoking question	To ascertain current, never and ex-smokers	Parts of the New Zealand Health Survey smoking panel Ministry of Health. 2015. Adult Questionnaire (Year 5) 1 July 2015 to 30 June 2016: New Zealand Health Survey. Wellington: Ministry of Health. Available from: http://www.health.govt.nz/publication/questionnaires-and-content-guide-2014-15-new-zealand-health-survey
Healthcare utilisation questions	Primary care, afterhours, Emergency Department ED and hospital admissions	Modified from the New Zealand Health Survey Ministry of Health. 2015. Adult Questionnaire (Year 5) 1 July 2015–30 June 2016: New Zealand Health Survey. Wellington: Ministry of Health. Available from: http://www.health.govt.nz/publication/questionnaires-and-content-guide-2014-15-new-zealand-health-survey
Comorbid conditions questions	List of conditions	List is a modified version of: Sanga O, Stucki G, Liang M, Fossel AH, Katz JN. The Self-Administered Comorbidity Questionnaire: a new method to assess comorbidity for clinical and health services research. *Arthritis Rheum* 2003; 49: 156–63. [62]
Medication questions	Name, dose and frequency	Questions developed by research team.
Health-related quality of life (EQ-5D-5 L)	Health profile and Visual Analogue Scale rating	Permission granted to use the paper and REDCap versions of the EQ-5D-5 L. Available from: https://euroqol.org/eq-5d-instruments/eq-5d-5l-about/
The summary of diabetes self-care activities	Original scale included 5 domains on self-care with additional items on what care has been recommended	We have focussed on the self-care domains only. We have kept the general categories outlined in the original scale, but have modified the questions on diet, blood sugar, foot checks, smoking and medications to be of use for our randomised controlled trial (RCT). Toobert DJ, Hampson SE, Glasgow RE. The summary of diabetes self-care activities measure: results from 7 studies and a revised scale. Diabetes care 23.7 (2000): 943–950. [38] Permission to use this scale is from: http://www.ori.org/sdsca/purchase.
Diabetes Distress Scale	17 questions on the level of distress resulting from diabetes	Permission granted to use the scale, and modify to also include pre-diabetics. Available from: http://behavioraldiabetes.org/scales-and-measures/#1448434304099-9078f27c-4106 Fisher L, Glasgow RE, Mullan JT, Skaff MM, Polonsky WH. Development of a brief diabetes distress screening instrument. *Ann Fam Med* 2008;6(3):246–252. [39] Polonsky WH, Fisher L, Esarles J, Dudl RJ, Lees J, Mullan JT, Jackson R. (2005). Assessing psychosocial distress in diabetes: Development of the Diabetes Distress Scale. Diabetes Care, 28, 626–631. [63] Fisher L, Hessler DM, Polonsky WH, Mullan J. (2012). When is diabetes distress clinically meaningful? Establishing cut-points for the Diabetes Distress Scale. Diabetes Care, 35, 259–264. [64]
Partners in Health Scale	12 questions on chronic disease self-management	Permission to use the scale, April 2017. Smith D, et al. Measuring chronic condition self-management in an Australian community: factor structure of the revised Partners in Health (PIH) scale. Quality of Life Research (2016), 1–11. [37] Petkov J, Harvey P, Battersby MW. The internal consistency and construct validity of the Partners in Health Scale: validation of a patient rated chronic condition self-management measure. Quality of Life Research 19.7 (2010), 1079–1085. [65]

### Intervention arm

BetaMe is Internet-based and delivered using both mobile and web-based platforms by Melon Health Ltd. [22]. The BetaMe intervention aims to strike a balance between ‘tailoring’ to individuals and delivering an intervention that is applied consistently enough to allow its impacts to be measured.

The BetaMe intervention consists of four separate components; health coaches, provision of evidence-based educational resources, goal tracking and peer support:

1. Health coaching. The health coaches are all either qualified personal trainers or registered nurses trained in motivational interviewing, cognitive behavioural therapy, and nutrition, and have completed the Heart Foundation Pacific Heartbeat Community Nutrition course. Health coaches arrange an initial one-on-one session with participants. The purpose of this first 1 h session is for health coaches to work with participants to develop goals tailored to their needs and values. The session follows a set format. Māori and Pacific coaches are available. Once these goals have been set and agreed on, participants’ goals are entered into their profile on the BetaMe system. Coaches will continue to work with participants, and will message participants weekly to check on their progress, reinforce information from the modules and arrange further meetings at frequencies that suit the participant (these can be weekly, fortnightly or less frequently if preferred). These subsequent meetings are generally around 15–20 min in length. All meetings with the coaches will be by audio or video, as per the participant’s preference. Support and clinical governance of the health coaches is provided by a clinical psychologist, general practitioner and an experienced health coach. Coaches also regularly interact with one another via an online forum (separate from patient forums) to share any problems they are having and solutions
2. Health literacy component. There are eight key education modules introduced to participants via the web and mobile platform with attached resources (videos, infographics, articles, tips, tools, meal ideas and mini-quiz). The key messages in the modules are reinforced in corresponding email newsletters, discussions on the newsfeed initiated by the community manager and by the participant’s coach. Every 2 weeks, a new module is released that presents participants with a range of topics that offer key messages to help them to learn about and manage their own health. The modules cover risk factors for T2DM and pre-diabetes, healthy eating and physical activity, identifying triggers for less healthy decisions and establishing healthy habits, sleep and shift work, meal planning, problem solving, healthy coping and mindfulness (see Additional file 2 for more information)
3. Goal tracking. Participants are encouraged to track their progress towards their goals (including healthy eating, physical activity, weight, happiness and sleep). They will be provided with a set of digital scales and a tape measure so they can take their own measurements and track their progress. They will be able to share these data with their health coach at any time, or with their physician by email or at their next appointment
4. Peer support. The programme also includes peer support with real-time chat within a private and secure online social network monitored by a community manager who is a registered nurse trained in nutrition. This person monitors the community to ensure nothing unsafe (like personal details, or negative health messages) is shared, and that people are supported and supportive. They will also reinforce key messages, highlight local events in the area, start conversations and welcome new people to the community. The community manager also provides a point of contact for both technical support, and for support more generally, particularly after the first 16 weeks of the programme when the health coaching module is completed.

In the first 16 weeks of the programme, participants will set goals, work with health coaches, receive the evidence-based modules and be encouraged to use goal tracking and participate in the peer support programme. After that time, participants will be encouraged to continue tracking and participating in peer support, for the remainder of the 52-week programme. Information from the modules is available for the entire 52-week period.

At the time of randomisation, contact details for participants randomised to the intervention arm will be forwarded to Melon Health Ltd. (with participant permission). A health coach will then contact the participant within five working days to initiate the programme. Those randomised to the intervention arm will begin the BetaMe programme whilst continuing to receive usual clinical care from their primary healthcare team. They will also receive resources produced and provided by Diabetes NZ.

#### Technical aspects of the mHealth platform

Melon Health’s platform is built to maximise patient confidentiality and general security of the application at all levels. The system exceeds New Zealand privacy requirements and meets US HIPAA (Health Insurance Portability and Accountability Act) security rules for health data, with regular auditing by external security specialists.

The platform currently integrates with over 350 wearables and biometric sensors through its open API (application programming interface), and platform is accessible through a web-based application or Android/iOS applications.

### Control arm

Participants in the control arm will receive usual care. Although this may vary somewhat between practices and GPs, the key interest is the extent to which the BetaMe programme may improve outcomes beyond that expected in the context of usual care. For people with T2DM, this would typically involve an annual diabetes review to monitor glycaemic control and assess the patient for the presence of any diabetes complications and a review of treatment. In addition, it could include education and advice on lifestyle factors, such as diet and exercise, provided by a trained practice nurse or diabetes specialist nurse. For people with pre-diabetes, usual care would typically include lifestyle advice and education about diet and exercise, and an annual HbA1c.

The study team will also provide participants in the control arm with standard information about pre-diabetes and T2DM and its management, from resources produced and provided by Diabetes NZ (Pamphlets entitled ‘Pre-diabetes’, ‘Diabetes and Healthy Food Choices’, and ‘Live Well with Diabetes’, available at https://www.diabetes.org.nz/pamphlet-ordering/). This is to increase retention of control arm participants in the study, and to ensure that all patients have basic information on T2DM and pre-diabetes.

### Randomisation and blinding

Based upon their baseline HbA1c test results (pre-diabetes or diabetes range), participants will be randomised into the intervention or control arm (1:1 allocation) using a computer-generated randomisation schedule stratified by participating practice and ethnicity (and separate for the two HbA1c-level groups). Randomisation will be completed within two working days of the completed baseline assessment. To ensure allocation concealment, randomisation will be centrally determined from within the REDCap electronic data capture platform *after* the patient is enrolled in the study (following the first assessment with the research nurse and when full eligibility based on current HbA1c level has been determined). To ensure balance in numbers allocated to each study arm, randomisation lists will be blocked.

The randomisation allocation list will not be visible to the team member(s) requesting the allocation, and this team member(s) will only have details on the patient ID, practice, and ethnicity when requesting allocation for any patient. The exact length of each randomisation block will be kept in a separate document held by the principal investigator (PI), project manager and project statistician, and thus will not be available to the team member(s) responsible for determining allocation.

Given the nature of the intervention, it is not possible to keep participants blind to their intervention status. Research nurses taking objective measurements will be kept blind to the intervention status of participants to the extent possible. However, when the nurse meets with the participant at the 4- and 12-month follow-up visits, it is not possible to guarantee that the nurses remain blind to the intervention allocation of the participant. The results dataset will be stored with the study arm identity blinded by code. The key to this code will be held independent of the analysis dataset by the PI, a second member of the research team (MM), and an independent member of the internal Data Monitoring Group. Data cleaning and analysis will be conducted by a statistician who is blind to group allocation. Once analysis is complete, the results will be unblinded.

### Outcome measures

The co-primary outcomes are changes in HbA1c and weight at 12 months. HbA1c provides the most sensitive measure of glycaemic control among those with T2DM. High levels are predictive of higher levels of complications and mortality. Weight (and waist circumference) are associated with T2DM, cardiovascular disease, several cancers and overall mortality [28–30]. We will focus on weight in kilograms as this is the most common outcome in systematic [31, 32] and Cochrane reviews, [33, 34] but for completeness we will also report differences in units of Body Mass Index (BMI).

Secondary outcomes are:

- Change in waist circumference at 12 months
- Change in blood pressure at 12 months. High blood pressure (common amongst people with diabetes) is associated with a higher risk of cardiovascular disease and mortality, and is amenable to lifestyle interventions [35, 36]

Additional self-reported outcomes include:

- Medications (regular prescription medications), and changes to medications and doses
- Changes in self-management of chronic conditions using the Partners in Health Scale. This is a validated tool for self-reported self-management of chronic conditions [37]
- Diabetes-specific behaviours and outcomes will be measured using two validated instruments: the Summary of Diabetes Self-Care Activities tool, which assesses participants’ self-care activities [38]; and the Diabetes Distress Scale which differentiates those with high and low levels of distress [39]
- Health-related quality of life measured using the five-level EuroQol five dimensions questionnaire (EQ-5D-5 L) [40]. Short-term outcomes: we will assess primary and secondary outcomes at 4 months. This will give an indication of the effect of the ‘active’ part of the intervention. Research has shown that even short-term decreases in HbA1c can have a long-term effect on health outcomes, with effects tending to attenuate over time [12, 13, 41]

Outcomes will be assessed for all participants (regardless of whether they complete the intervention) by trained nurses at baseline, 4 months and 12 months (Tables 2 and 3).

### Sample size and analysis

All primary analyses will be based on intention-to-treat principles (with individuals analysed in the group to which they were randomised). Demographic and baseline characteristics will be summarised using descriptive statistics. Both HbA1c and weight will be analysed as continuous variables. For the HbA1c primary outcome, analysis will be conducted both combined and separately for pre-diabetes and T2DM. For weight, the primary analysis will combine participants with pre-diabetes and T2DM. Analysis will use linear mixed models, comparing mean outcome levels between intervention and control arms at the 12-month endpoint, adjusted for initial HbA1c level (accounting for baseline differences in outcome) and for other important baseline covariates (age, gender, ethnicity, weight). This analysis will include the 4-month measurements, providing some information for those individuals subsequently lost to follow-up and hence missing final outcome data (mixed models analysis treats these final data as missing at random conditional on baseline and intermediate covariates, i.e. these individuals are expected on average to have final outcomes similar to other people with the same baseline covariates and HbA1c trajectories). As the study involves repeated measures data, these models will include random effects for individuals to account for correlation between measurements from the same person at different follow-up times.

The study is powered in the pre-diabetes group to detect a difference of 2.5 mmol/mol between study arms (80% power, alpha = 0.05, presuming SD = 5.5 mmol/mol [42]). This requires a sample size of 76 per study arm (152 total). In the pilot study, the mean reduction in HbA1c was 2.6 mmol/mol. The sample size for the T2DM group is powered to detect a minimal clinically important difference [43, 44] of 5.5 mmol/mol between study arms (80% power, alpha for comparison = 0.05, presuming a standard deviation of 15 mmol/mol in this group [16]). This requires a sample size of 117 per study arm (234 total). This gives a combined sample size of 386 people with pre-diabetes and people with T2DM. To account for loss of precision due to patients lost to follow-up (projected at 10% [45, 46]), we will recruit 430 patients at baseline (note that all participants will be included in linear mixed models analysis, even if missing final endpoint data, which reduces bias in estimating effect size, but there is still loss in statistical power compared to having complete follow-up data).

Analysis of weight differences between study arms will use the same analytical methods as for the HbA1c outcome. For these weight analyses, those with pre-diabetes and T2DM will be pooled prior to analysis as the relevant intervention target weight loss is the same for these two groups. Analysis will be stratified on patient group to account for the pooled approach, and we will also report weight differences by study arm within each patient group. The comparison of weight loss in the combined patient group is powered to detect a difference between study arms of 5 kg (80% power, alpha = 0.05, SD = 15 kg in this group [47]). This requires a sample size of 142 per study arm (284 total), which is within the combined sample size calculated for the HbA1c outcome.

We will also estimate differences in HbA1c and weight endpoints by study arm for each of the stratified randomisation ethnic groups (Māori, Pacific, other ethnicities), and will report the mean intervention effect and 95% confidence interval for each group. Proportions of patients meeting HbA1c and weight-loss targets will also be analysed by ethnic group with logistic regression (adjusted for baseline covariates), and adjusted absolute risk differences in treatment outcomes will be computed to quantify treatment effectiveness [48]. Assuming that 20% of the sample will be Māori (*n* = 234 × 0.2 = 46 Māori participants) gives an anticipated margin of error on the intervention effect of ± 4.5 mmol/mol for HbA1c in the diabetic group.

Analysis of self-reported outcomes will be conducted in a similar way.

Results will be actively disseminated (see Additional file 3).

#### Data management

Baseline and follow-up data are entered directly by patients and research nurses at the time of the relevant assessments in to a REDCap database. Range checks are in place for relevant variables such as HbA1c levels (41–70 mmol/mol). All data are checked by research nurses at the time of their entry and those variables that are used for stratified randomisation (ethnicity, practice and HbA1c) are re-checked by the research manager prior to randomisation. Participants’ data will be de-identified by a unique code number and information linking that code to a particular patient (NHI and unique trial-specific code number) will be kept in a secure location with password protection. A master list which links the patients’ NHI and trial code number will be kept so any information about the study, including results, can be communicated to participants. Analysis will be carried out on de-identified data. All trial-specific data for the study will be securely stored by the research team for 10 years once the trial has ended.

#### Data monitoring

An independent Data Monitoring Group is not required for this study because the study is low risk in terms of likelihood of life-threatening complications or serious adverse events, the study team has no professional or financial links with the company that is delivering the intervention, and it will not be necessary to instigate stopping rules because there will be no interim analyses of efficacy [49]. An internal Data Monitoring Group will be convened at 2-monthly intervals which will consist of the study PIs (DS and MM) and the biostatistician (JS). This group will also include an independent senior researcher. This group will assess the effectiveness of study procedures, review any potential adverse events and review and approve any amendments to study protocols. Other members of the research group will provide advice and input if requested.

### Process evaluation

At the end of the study, there will be a process evaluation, which will include assessments of implementation of the BetaMe programme and mechanisms of action. Data will be collected from three sources: (1) from the mobile/web platform to identify key usage patterns; (2) with consent from the participants, health coaches will record audio interactions with participants and will log key reasons for contact (a random subset of the coaching sessions will be assessed by the research team for consistency with the log); and (3) participants will be invited to provide individual feedback on the acceptability and usefulness of the intervention at the end of the study period.

Focus groups, lasting approximately 1 h each, will be held in each region to obtain in-depth feedback about the intervention and how participants interacted with it. Usage will be described, and linked to outcomes using standard quantitative analytic approaches (for example, proportions accessing health coaches will be reported, with 95% confidence intervals; frequency of accessing health coaches or engaging online will be reported as median access with range/interquartile range). Focus group data will be coded thematically, with key issues identified.

### Trial registration

The trial is registered with the Australian New Zealand Clinical Trials Registry on 19 April 2017. Registration number: ACTRN: ACTRN12617000549325. See http://www.ANZCTR.org.au/ACTRN12617000549325.aspx. The full protocol is available at this site, including consent forms and detailed documentation. Any important protocol modifications will be reported to this organisation.

This trial’s Universal Trial Number (UTN) is U1111–1189-9094.

## Discussion

Long-term conditions pose a great burden to patients, family/whānau and health services. Self-management interventions are a potential way to address this burden with evidence that they can be effective in improving physical and mental health. Important gaps in evidence remain, and are a barrier to implementation. The BetaMe programme is an integrated, evidence-based, comprehensive, self-management programme delivered by mobile and web-based technology. A pilot of 117 patients with pre-diabetes was extremely successful with 78% of people reducing their HbA1c level below pre-diabetes range over 4 months. However, the study was volunteer-based and did not incorporate a comparison arm.

This paper describes the protocol of a study that will address the question of whether this kind of programme is likely to be effective in reducing HbA1c and weight among people with T2DM and pre-diabetes in a primary care environment. It addresses shortcomings of previous studies and includes an explicit aim to assess the effectiveness of this kind of programme among Māori and Pacific people who have higher rates of T2DM and pre-diabetes than other ethnic groups in NZ.

If found to be effective, this programme would allow healthcare providers to deliver an intervention that is person-centred and supports the self-care of people with T2DM, pre-diabetes and potentially other LTCs.

## Trial status

This paper is based on protocol version 1.2, dated 1 August 2017. Recruitment to the study began on 9 June 2017. Approximate date of recruitment completion: February 2018.

## Additional files

Additional file 1: SPIRIT Checklist. (DOC 120 kb)

Additional file 2: Health literacy modules. (DOCX 14 kb)

Additional file 3: Dissemination policy. (DOCX 18 kb)

## Abbreviations

BMI: Body Mass Index
EQ-5D-5L: five-level EuroQol five dimensions questionnaire
LTC: Long-term conditions
NHI: National Health Index
NZ: New Zealand
PHO: Primary Healthcare Organisation
PI: Primary investigator
T2DM: Type 2 diabetes mellitus

## References

[1] Ministry of Health (2013). Health Loss in New Zealand: A report from the New Zealand Burden of Diseases, Injuries and Risk Factors Study, 2006–2016.

[2] Ministry of Health (2015). Living Well with Diabetes: a plan for people at high risk of or living with diabetes 2015–2020.

[3] Coppell KJ, Mann JI, Williams SM, Jo E, Drury PL, Miller J, Parnell WR (2013). Prevalence of diagnosed and undiagnosed diabetes and prediabetes in New Zealand: findings from the 2008/09 Adult Nutrition Survey. NZ Med J.

[4] World Health Organization (2006). Definition and diagnosis of diabetes mellitus and intermediate hyperglycemia: report of a WHO/IDF consultation.

[5] Cheng YJ, Gregg EW, Geiss LS, Imperatore G, Williams DE, Zhang X, Albright AL, Cowie CC, Klein R, Saaddine JB (2009). Association of A1C and fasting plasma glucose levels with diabetic retinopathy prevalence in the U.S. population: implications for diabetes diagnostic thresholds. Diabetes Care.

[6] Diabetes Prevention Program Research G (2007). The prevalence of retinopathy in impaired glucose tolerance and recent-onset diabetes in the Diabetes Prevention Program. Diabet Med.

[7] Ford ES, Zhao G, Li C (2010). Pre-diabetes and the risk for cardiovascular disease: a systematic review of the evidence. J Am Coll Cardiol.

[8] Ziegler D, Rathmann W, Dickhaus T, Meisinger C, Mielck A, Group KS (2008). Prevalence of polyneuropathy in pre-diabetes and diabetes is associated with abdominal obesity and macroangiopathy: the MONICA/KORA Augsburg Surveys S2 and S3. Diabetes Care.

[9] Vinik AI, Richardson DW (1997). Implications of the diabetes control and complications trial for persons with non-insulin-dependent diabetes mellitus. South Med J.

[10] Knowler WC, Fowler SE, Hamman RF, Christophi CA, Hoffman HJ, Brenneman AT, Brown-Friday JO, Goldberg R, Venditti E, Diabetes Prevention Program Research G (2009). 10-year follow-up of diabetes incidence and weight loss in the Diabetes Prevention Program Outcomes Study. Lancet.

[11] Lindstrom J, Ilanne-Parikka P, Peltonen M, Aunola S, Eriksson JG, Hemio K, Hamalainen H, Harkonen P, Keinanen-Kiukaanniemi S, Laakso M (2006). Sustained reduction in the incidence of type 2 diabetes by lifestyle intervention: follow-up of the Finnish Diabetes Prevention Study. Lancet.

[12] Perreault L, Pan Q, Mather KJ, Watson KE, Hamman RF, Kahn SE (2012). Diabetes Prevention Program Research G: effect of regression from prediabetes to normal glucose regulation on long-term reduction in diabetes risk: results from the Diabetes Prevention Program Outcomes Study. Lancet.

[13] Knowler WC, Barrett-Connor E, Fowler SE, Hamman RF, Lachin JM, Walker EA, Nathan DM (2002). Diabetes Prevention Program Research G: reduction in the incidence of type 2 diabetes with lifestyle intervention or metformin. N Engl J Med.

[14] Rivellese AA, Boemi M, Cavalot F, Costagliola L, De Feo P, Miccoli R, Patti L, Trovati M, Vaccaro O, Zavaroni I (2008). Dietary habits in type II diabetes mellitus: how is adherence to dietary recommendations?. Eur J Clin Nutr.

[15] Ruggiero L, Glasgow R, Dryfoos JM, Rossi JS, Prochaska JO, Orleans CT, Prokhorov AV, Rossi SR, Greene GW, Reed GR (1997). Diabetes self-management. Self-reported recommendations and patterns in a large population. Diabetes Care.

[16] Liang X, Wang Q, Yang X, Cao J, Chen J, Mo X, Huang J, Wang L, Gu D (2011). Effect of mobile phone intervention for diabetes on glycaemic control: a meta-analysis. Diabet Med.

[17] Nundy S, Dick JJ, Chou CH, Nocon RS, Chin MH, Peek ME (2014). Mobile phone diabetes project led to improved glycemic control and net savings for Chicago plan participants. Health Aff.

[18] Norris SL, Engelgau MM, Narayan KM (2001). Effectiveness of self-management training in type 2 diabetes: a systematic review of randomized controlled trials. Diabetes Care.

[19] Franklin VL, Waller A, Pagliari C, Greene SA (2006). A randomized controlled trial of Sweet Talk, a text-messaging system to support young people with diabetes. Diabet Med.

[20] Wolever RQ, Dreusicke MH (2016). Integrative health coaching: a behavior skills approach that improves HbA1c and pharmacy claims-derived medication adherence. BMJ Open Diabetes Res Care.

[21] Sepah SC, Jiang L, Peters AL (2015). Long-term outcomes of a web-based diabetes prevention program: 2-year results of a single-arm longitudinal study. J Med Internet Res.

[22] Melon Health Ltd. http://www.melonhealth.com/. Accessed on 10 Jan 2017.

[23] Abraham C, Michie S (2008). A taxonomy of behavior change techniques used in interventions. Health Psychol.

[24] Ryan P (2009). Integrated Theory of Health Behavior Change: background and intervention development. Clin Nurse Spec.

[25] Bulfin S, Newell S. BetaMe-digital prediabetes self-management pilot. In: Health Informatives NZ Conference: 2016; Auckland; 2016. p. 25. https://c.ymcdn.com/sites/hinz.site-ym.com/resource/resmgr/conference_2016/Proceedings/HiNZ2016_Proceedings_for_USB.pdf.

[26] Nobel J, Sasser E (2016). Digital diabetes prevention and management solutions.

[27] Tomlin A, Sinclair A (2016). The influence of cognition on self-management of type 2 diabetes in older people. Psychol Res Behav Manag.

[28] Klein S, Allison DB, Heymsfield SB, Kelley DE, Leibel RL, Nonas C, Kahn R, Association for Weight M, Obesity P, Naaso TOS (2007). Waist circumference and cardiometabolic risk: a consensus statement from Shaping America’s Health: Association for Weight Management and Obesity Prevention; NAASO, The Obesity Society; the American Society for Nutrition; and the American Diabetes Association. Am J Clin Nutr.

[29] Price GM, Uauy R, Breeze E, Bulpitt CJ, Fletcher AE (2006). Weight, shape, and mortality risk in older persons: elevated waist-hip ratio, not high body mass index, is associated with a greater risk of death. Am J Clin Nutr.

[30] Blakely T, Sarfati D, Shaw C (2009). What proportion of cancer is due to obesity?. NZ Med J.

[31] Greaves CJ, Sheppard KE, Abraham C, Hardeman W, Roden M, Evans PH, Schwarz P, Group IS (2011). Systematic review of reviews of intervention components associated with increased effectiveness in dietary and physical activity interventions. BMC Public Health.

[32] Terranova CO, Brakenridge CL, Lawler SP, Eakin EG, Reeves MM (2015). Effectiveness of lifestyle-based weight loss interventions for adults with type 2 diabetes: a systematic review and meta-analysis. Diabetes Obes Metab.

[33] Norris SL, Zhang X, Avenell A, Gregg E, Schmid CH, Lau J (2005). Long-term non-pharmacological weight loss interventions for adults with prediabetes. Cochrane Database Syst Rev.

[34] Wieland LS, Falzon L, Sciamanna CN, Trudeau KJ, Brodney S, Schwartz JE, Davidson KW (2012). Interactive computer-based interventions for weight loss or weight maintenance in overweight or obese people. Cochrane Database Syst Rev.

[35] Blumenthal JA, Sherwood A, Gullette EC, Babyak M, Waugh R, Georgiades A, Craighead LW, Tweedy D, Feinglos M, Appelbaum M (2000). Exercise and weight loss reduce blood pressure in men and women with mild hypertension: effects on cardiovascular, metabolic, and hemodynamic functioning. Arch Intern Med.

[36] Maruthur NM, Wang NY, Appel LJ (2009). Lifestyle interventions reduce coronary heart disease risk: results from the PREMIER Trial. Circulation.

[37] Smith D, Harvey P, Lawn S, Harris M, Battersby M (2017). Measuring chronic condition self-management in an Australian community: factor structure of the revised Partners in Health (PIH) scale. Qual Life Res.

[38] Toobert DJ, Hampson SE, Glasgow RE (2000). The summary of diabetes self-care activities measure: results from 7 studies and a revised scale. Diabetes Care.

[39] Fisher L, Glasgow RE, Mullan JT, Skaff MM, Polonsky WH (2008). Development of a brief diabetes distress screening instrument. Ann Fam Med.

[40] Herdman M, Gudex C, Lloyd A, Janssen M, Kind P, Parkin D, Bonsel G, Badia X (2011). Development and preliminary testing of the new five-level version of EQ-5D (EQ-5D-5L). Qual Life Res.

[41] Pal K, Eastwood SV, Michie S, Farmer AJ, Barnard ML, Peacock R, Wood B, Inniss JD, Murray E (2013). Computer-based diabetes self-management interventions for adults with type 2 diabetes mellitus. Cochrane Database Syst Rev.

[42] Garvey WT, Ryan DH, Henry R, Bohannon NJ, Toplak H, Schwiers M, Troupin B, Day WW (2014). Prevention of type 2 diabetes in subjects with prediabetes and metabolic syndrome treated with phentermine and topiramate extended release. Diabetes Care.

[43] US Food and Drug Administration. Guidance for industry: Diabetes Mellitus: Developing Drugs and Therapeutic Biologics for Treatment and Prevention. Rockville: US Food and Drug Administration; 2008.

[44] Mearns ES, Sobieraj DM, White CM, Saulsberry WJ, Kohn CG, Doleh Y, Zaccaro E, Coleman CI (2015). Comparative efficacy and safety of antidiabetic drug regimens added to metformin monotherapy in patients with type 2 diabetes: a network meta-analysis. PLoS One.

[45] Cho JH, Lee HC, Lim DJ, Kwon HS, Yoon KH (2009). Mobile communication using a mobile phone with a glucometer for glucose control in Type 2 patients with diabetes: as effective as an Internet-based glucose monitoring system. J Telemed Telecare.

[46] Quinn CC, Shardell MD, Terrin ML, Barr EA, Ballew SH, Gruber-Baldini AL (2011). Cluster-randomized trial of a mobile phone personalized behavioral intervention for blood glucose control. Diabetes Care.

[47] Heymsfield SB, Segal KR, Hauptman J, Lucas CP, Boldrin MN, Rissanen A, Wilding JPH, Sjöström L (2000). Effects of weight loss with Orlistat on glucose tolerance and progression to type 2 diabetes in obese adults. Arch Intern Med.

[48] Muller CJ, MacLehose RF (2014). Estimating predicted probabilities from logistic regression: different methods correspond to different target populations. Int J Epidemiol.

[49] Health Research Council of New Zealand. Data Monitoring Core Committee. http://www.hrc.govt.nz/ethics-and-regulatory/data-monitoring-core-committee#what-appropriate. Accessed on 16 Jan 2017.

[50] Funnell MM, Brown TL, Childs BP, Haas LB, Hosey GM, Jensen B, Maryniuk M, Peyrot M, Piette JD, Reader D (2009). National standards for diabetes self-management education. Diabetes Care.

[51] Clarke DM, Baird DE, Perera DN, Hagger VL, Teede HJ (2014). The INSPIRED study: a randomised controlled trial of the Whole Person Model of disease self-management for people with type 2 diabetes. BMC Public Health.

[52] Jordan JE, Osborne RH (2007). Chronic disease self-management education programs: challenges ahead. Med J Austr.

[53] Cole-Lewis H, Kershaw T (2010). Text messaging as a tool for behavior change in disease prevention and management. Epidemiol Rev.

[54] Fischer HH, Fischer IP, Pereira RI, Furniss AL, Rozwadowski JM, Moore SL, Durfee MJ, Raghunath SG, Tsai AG, Havranek EP (2016). Text message support for weight loss in patients with prediabetes: a randomized clinical trial. Diabetes Care.

[55] Yamada T, Kiuchi Y, Nemoto M, Yamashita S (2014). Charting weight four times daily as an effective behavioural approach to obesity in patients with type 2 diabetes. Diab Vasc Dis Res.

[56] Glasgow RE, Funnell MM, Bonomi AE, Davis C, Beckham V, Wagner EH (2002). Self-management aspects of the improving chronic illness care breakthrough series: implementation with diabetes and heart failure teams. Ann Behav Med.

[57] Jennings A, Powell J, Armstrong N, Sturt J, Dale J (2009). A virtual clinic for diabetes self-management: pilot study. J Med Internet Res.

[58] Thom DH, Ghorob A, Hessler D, De Vore D, Chen E, Bodenheimer TA (2013). Impact of peer health coaching on glycemic control in low-income patients with diabetes: a randomized controlled trial. Ann Fam Med.

[59] Fisher EB, Boothroyd RI, Coufal MM, Baumann LC, Mbanya JC, Rotheram-Borus MJ, Sanguanprasit B, Tanasugarn C (2012). Peer support for self-management of diabetes improved outcomes in international settings. Health Aff.

[60] Yin J, Wong R, Au S, Chung H, Lau M, Lin L, Tsang C, Lau K, Ozaki R, So W (2015). Effects of providing peer support on diabetes management in people with type 2 diabetes. Ann Fam Med.

[61] Vissenberg C, Stronks K, Nijpels G, Uitewaal PJ, Middelkoop BJ, Kohinor MJ, Hartman MA, Nierkens V (2016). Impact of a social network-based intervention promoting diabetes self-management in socioeconomically deprived patients: a qualitative evaluation of the intervention strategies. BMJ Open.

[62] Sangha O, Stucki G, Liang MH, Fossel AH, Katz JN (2003). The Self-Administered Comorbidity Questionnaire: a new method to assess comorbidity for clinical and health services research. Arthritis Rheum.

[63] Polonsky WH, Fisher L, Earles J, Dudl RJ, Lees J, Mullan J, Jackson RA (2005). Assessing psychosocial distress in diabetes: development of the diabetes distress scale. Diabetes Care.

[64] Fisher L, Hessler DM, Polonsky WH, Mullan J (2012). When is diabetes distress clinically meaningful?: establishing cut points for the Diabetes Distress Scale. Diabetes Care.

[65] Petkov J, Harvey P, Battersby M (2010). The internal consistency and construct validity of the Partners in Health Scale: validation of a patient rated chronic condition self-management measure. Qual Life Res.

