# Supplementary material for: BetaMe: impact of a comprehensive digital health programme on HbA1c and weight at 12 months for people with diabetes and pre-diabetes: study protocol for a randomised controlled trial
Source: Trials. 2018 Mar 5;19:161. doi: 10.1186/s13063-018-2528-4 (PMC5836439; doi:10.1186/s13063-018-2528-4)
Supplement: Supplementary file 2 — Health literacy modules. (DOCX 14 kb) [file 13063_2018_2528_MOESM2_ESM.docx]

**Additional file 2: Health literacy modules of BetaMe intervention**

There are eight key education modules delivered to participants through the web and mobiles with a variety of resources including videos, infographics, articles, tips, tools, meal ideas and mini-quiz. New modules are released every two weeks for the first 16 weeks of the BetaMe programme. Each offers key messages to help participants to learn about and manage their own health. The modules are:

**Week 0**: Introduction to BetaMe and risk factors of prediabetes and Diabetes. This module includes an overview of what prediabetes and diabetes are in plain language including risk factors, signs and symptoms, what lifestyle factors can prevent or modify diabetes and prediabetes. There is also an introduction to physical activity, the food diary and tracking.

**Week 1-2**: Healthy Eating, Healthy Moving. This module provides support to participants to change their eating and physical activity patterns. It encourages them to get support and encouragement from the BetaMe community, and offers other techniques including visualisation, meditation and goal setting.

**Week 3-4**: Triggers and Habits. This module encourages participants to identify when and why they make less healthy decisions, and how to establish healthy habits. It reinforces the importance of tracking.

**Week 5**: Review your progress. This module offers a quiz on key learning points about healthy eating and activity.

**Week 6-7**: Eating for Health. This module identifies cheap, convenient and tasty meal ideas, and reinforces the importance of exercise.

**Week 8-9**: Drinks, Sleep and Shift Work. This module identifies alternatives to sugary drinks, and focuses on the importance of sleep.

**Week 10**: Recap & Refresh, Meal Planning and Budgeting Tips.

**Week 11-12**: Problem Solving. This module provides participants with skills and confidence to make informed decisions about food and activity.

**Week 13-14**: Healthy Coping & Mindfulness. This module identifies self-defeating thoughts and provides strategies on how to break them including breathing exercises, affirmations, mindfulness and visualisation.

**Week 15-16**: Reflection and Celebration. Transitioning to the maintenance phase. This module reinforces key messages, provides a quiz, and the process to identify next steps to ongoing success.

The content of these modules aligns with the Ministry of Health Guidelines for health eating and the Heart Foundation guidelines.[1, 2] Participants will have the option for one-on-one on-line chats with their coach to discuss each new module.

1. Ministry of Health, *Eating and Activity Guidelines for New Zealand Adults*. 2015, Ministry of Health: Wellington.

2. Heart Foundation of New Zealand, *The Healthy Heart: A Guideline for Health Professionals*, H.F.o.N. Zealand, Editor. 2013.
