# Supplementary material for: BetaMe: impact of a comprehensive digital health programme on HbA1c and weight at 12 months for people with diabetes and pre-diabetes: study protocol for a randomised controlled trial
Source: Trials. 2018 Mar 5;19:161. doi: 10.1186/s13063-018-2528-4 (PMC5836439; doi:10.1186/s13063-018-2528-4)
Supplement: Supplementary file 3 — Dissemination policy. (DOCX 18 kb) [file 13063_2018_2528_MOESM3_ESM.docx]

**Additional file 3: Dissemination plan for BetaMe study**

#### Dissemination strategy

The study team has an explicit translation and dissemination strategy which includes the following steps:

**Content of evidence to translate:** the likelihood of successful research translation is increased if it is consistent with a recognised clinical need and is applicable to local contexts.[1, 2] This proposal has been developed with input from key stakeholders and they are committed to both supporting the evaluative aspects and directly translating the findings widely.

**Strategies**: our process evaluation will identify barriers and facilitators to translation, and we will actively engage with our key stakeholders to identify specific strategies to optimise translation of the findings. Without pre-empting what these strategies will eventually be, it is anticipated that they will include the following;

- 1. **Patient and whanau/family strategies**- We will provide summaries of research findings directly to participants. We will also promote research findings through relevant patient support networks, develop lay summaries of research and promotion material for distribution to primary care practices and other community organisations. We will actively seek out opportunities to feedback findings to key Māori and Pacific health groups such as Te ORA (Māori Medical Practitioner’s Association) and the Pasifika Medical Association. Press releases will also be made available to Māori media. A summary of research findings will be translated into Te Reo Māori.
  2. **Community-based organisations**- We will provide research summaries and presentations to community organisations such as Diabetes NZ, and to Māori and Pacific communities.
  3. **Healthcare practitioner strategies**- We will work with the partner PHO’s to present the findings of what is an innovative new solution to supporting patient self-management in a way that resonates with frontline practitioners and addresses their concerns. We will seek opportunities to present this work at conferences and within relevant media (e.g NZ Doctor).
  4. **Healthcare administrators**- We have excellent levels of support from Compass and Midlands Health Network. Both have indicated a commitment to promoting the findings within their networks of practices. They also have strong linkages with NZ’s two other large PHO’s (ProCare and Pegasus). This network of four PHOs have an established process of sharing innovative models of care and would immediately expose the research findings to a network of practices accounting for around half of NZ’s population.
  5. **Policy Strategies**- There is currently strong policy support at a central government level for innovative solutions to support patient centred care (updated NZ Health Strategy). We will seek to present research findings to relevant groups within DHBs and the Ministry of Health.
  6. **Other strategies**- In addition to these strategies, we will publish and present results in academic peer-reviewed papers, at academic and clinical conferences, and in ‘popular medical press’. Key publications will be accompanied by media releases. Authorship eligibility will be on the basis of the Vancouver criteria. Neither the funders nor Melon Health Ltd have any role in the content of publications, nor on the decision to publish. Publications are provided to the Health Research Council prior to publication for their information.

1. Boaz A, Baeza J, Fraser A: **Effective Implementation of research into practice: an overview of systematic reviews of the health literature**. *BMC Res Notes* 2011, **4**:212.

2. Kitson A, Harvey G, McCormack B: **Enabling the implementation of evidence based practice: a conceptual framework**. *Qual Health Care* 1998, **7**(3):149-158.
